# Impact of Different Lactic Acid Bacteria on the Properties of Rice Sourdough and the Quality of Steamed Rice Bread

**DOI:** 10.3390/foods14244335

**Published:** 2025-12-16

**Authors:** Jiaqi Lin, Lijia Dong, Xueyuan Han, Jianqiu Sun, Chi Shen, Huanyi Yang

**Affiliations:** School of Life and Environmental Science, Shaoxing University, Shaoxing 312000, China; ljq2733744166@163.com (J.L.); donglijia@126.com (L.D.); 18867146394@163.com (X.H.); jianqius@163.com (J.S.); sc@usx.edu.cn (C.S.)

**Keywords:** lactic acid bacteria, sourdough, rice, gluten-free, steamed bread

## Abstract

The influence of lactic acid bacteria (LAB) strains of various species isolated from Chinese traditional sourdough on the properties of rice sourdough and the textural and flavor qualities of steamed rice bread (SRB) was investigated. *Lactiplantibacillus plantarum*-fermented rice sourdough had a higher total titratable acidity (13.10 mL) than the other groups. Strains *Lacticaseibacillus paracasei* PC1 (LPC), *Lactobacillus helveticus* H1 (LH), *Lactobacillus crustorum* C1 (LC), *Lactobacillus paralimentarius* PA1 (LPA), and *Lactiplantibacillus plantarum* P1 (LP) showed marked protein hydrolysis during rice sourdough fermentation and increased free amino acid levels in rice sourdoughs relative to the control. The Fourier Transform Infrared Spectroscopy results indicated that LAB fermentation could promote the strengthening of inter-intramolecular hydrogen bonds and cause modifications in protein structures; however, these effects varied among the different strains. The LC and LPC strains had the most significant effect on improving the specific volume and textural properties of SRBs. Gas chromatography-mass spectrometry (GC-MS) and GC-ion mobility spectrometry (IMS) identified 33 and 35 volatile compounds, respectively, in the LAB-fermented SRBs, and differentiation was observed in the volatile profiles of SRBs made using different LAB strains. The differential impacts of LAB strains during rice sourdough fermentation can assist in the selection of candidate microorganisms for the production of high-quality gluten-free rice products.

## 1. Introduction

The incidence of gluten-related diseases, particularly celiac disease (CD), has increased substantially in recent years, thereby augmenting the need for gluten-free (GF) products. CD is an inflammatory disorder of the small intestine that affects individuals who are intolerant to gluten from rye, barley, wheat, and their crossbred varieties [1]. Currently, CD treatment involves a lifelong, rigorous GF diet to prevent the immunological reactions that gluten causes [2].

Rice is among the most frequently utilized GF foods because it contains no gluten protein, is known for being hypoallergenic, and is easy to digest. In addition, rice is low in fiber, fat, and sodium [3]. However, the absence of gluten hinders network formation in the dough when making bread or steamed bread, leading to final products with reduced volume, firm texture, and poor sensory qualities [4]. Therefore, researchers have aimed to improve the characteristics of GF rice products by incorporating non-gluten proteins, hydrocolloids, or transglutaminase [5,6,7,8]. Nevertheless, the use of these additives is not ideal because they have been linked to problems, such as excessive ingredient costs and the potential to induce allergies [9]. The increasing demand for natural, clean-label foods has driven the need for innovative methods to produce GF products.

Sourdough is a mixture of flour and water fermented using lactic acid bacteria (LABs) and yeast. It has been used for thousands of years to produce cereal-based fermented products [10]. Previous research on GF products indicated that the application of the sourdough technique yields beneficial outcomes by enhancing volume, texture, flavor, and nutritional content, which are attributed to the intrinsic metabolic processes of LAB and yeasts [11,12]. LAB in sourdough can produce organic acids and exopolysaccharides (EPS) to improve dough rheology, bread texture, and flavor by producing various volatile compounds [13]. Furthermore, acidification caused by LAB strains can stimulate endogenous amylases and proteases, and the hydrolysis of starch and proteins improves the water retention of dough by enhancing its water-binding capacity and solubility, resulting in a softer bread crumb texture [14,15]. Therefore, sourdough can be used to resolve problems related to the production of low-quality GF products and replace expensive additives to improve GF cereal-based products because of their low cost and environmental friendliness.

Steamed bread has been a staple food in China for more than 1500 years and has become the most popular traditional fermented product among the Chinese since it is simple to prepare and consume [16]. In addition, steamed bread is also considered to be more nutritionally advantageous than bread due to its minimal nutritional loss during steaming. Sourdough is traditionally served as the starter for steamed bread preparation and is still preferred nowadays for its particular taste. During the sourdough fermentation, the LAB produce metabolites such as organic acids, EPS, and a variety of volatile compounds, thus improving the specific volume, texture, and flavor of steamed bread [17,18].

Although improving the quality of rice-based products using sourdough has been reported, few studies have thoroughly explored the role of different LAB strains during rice sourdough fermentation and their effects on rice sourdough properties and steamed rice bread (SRB) quality. This study aimed to elucidate the impact of different LAB strains isolated from Chinese traditional sourdoughs on the properties of rice sourdough and the textural and flavor qualities of SRB, thereby evaluating the potential application of LAB for the production of high-quality GF rice products.

## 2. Materials and Methods

### 2.1. Microbial Strains

Eight LAB strains of different species isolated from Chinese traditional sourdough were used in this study: *Lactobacillus helveticus* H1 (LH), *Lactobacillus paralimentarius* PA1 (LPA), *Lactobacillus brevis* B1 (LB), *Lacticaseibacillus paracasei* PC1 (LPC), *Lactobacillus crustorum* C1 (LC), *Lactiplantibacillus plantarum* P1 (LP), *Lactobacillus mindensis* M1 (LM), and *Fructilactobacillus sanfranciscensis* S1 (FS). The isolation and identification of these strains were previously described in a study by Liu et al. [19]. FS and LM were incubated in SDB broth [20], whereas De Man, Rogosa, and Sharpe (MRS) broth (Hangzhou Microbial Reagent Co., Ltd., Hangzhou, China) was used to cultivate the other LAB strains. All strains used in the experiments were stored at −80 °C in the respective broth augmented with 25% (*v*/*v*) glycerol.

### 2.2. Rice Sourdough Fermentation

Rice flour was procured from Henan Mugu Biotechnology Co., Ltd. (Zhengzhou, China) and contained 78.3% carbohydrates, 5.8% protein, and 1.3% fat. Each LAB strain was anaerobically incubated in SDB or MRS broth at 30 °C to late-log phase. The centrifugation of the cells was carried out at 4000× *g* and 4 °C for 15 min, followed by rinsing twice with sterile distilled water as previously described by Zhang et al. [21]. The cells were resuspended in sterile water at a 10^8^ colony-forming units (CFU)/mL concentration and protected by their cell walls from lysing due to changes in osmotic pressure. The LAB suspension was then mixed with rice flour in a 1:1 ratio. Each dough sample was individually inoculated with different LAB strains in triplicate. The control group received the same volume of sterile water as the LAB suspension group. The doughs were then kept at 30 °C and 80% relative humidity for 12 h. The rice sourdoughs were either immediately processed to determine the pH and total titratable acidity (TTA) or freeze-dried for subsequent analysis.

### 2.3. pH and TTA Analysis

The pH and TTA of rice sourdoughs were assessed as previously described [22]. Each sample (10 g) was mixed with distilled water (90 mL) and homogenized for 5 min. A pH meter (FE28, Mettler Toledo, Greifensee, Switzerland) was used for pH analysis. The measurement of TTA was carried out through titration and was indicated as the volume of 0.1 mol/L NaOH needed to achieve a pH value of 8.5.

### 2.4. Sodium Dodecyl Sulfate—Polyacrylamide Gel Electrophoresis (SDS-PAGE)

The freeze-dried sourdough powder (50 mg) was suspended in 0.3 mL of distilled water and 0.1 mL SDS-PAGE loading buffer (4× with β-mercaptoethanol; Solarbio Science & Technology Co., Ltd., Beijing, China). Suspensions were heated for 5 min at 100 °C, followed by centrifugation for 10 min at 12,000× *g* to collect supernatant, 10 μL of which was subjected to electrophoresis on a discontinuous gel system consisting of 10% separating and 5% stacking gels. The electrophoresis was conducted at 100 V. The gel was stained with Coomassie Brilliant Blue R-250 (0.25%, *w*/*v*) and destained in a washing buffer containing 40% methanol (*v*/*v*) and 10% acetic acid (*v*/*v*), (Imperial Chemical Industries, London, UK).

### 2.5. Fourier Transform Infrared (FT-IR) Spectroscopy Analysis

The FT-IR profiles of rice sourdoughs were determined using an FT-IR spectrometer (Nicolet iS 20, Thermo Scientific, Waltham, MA, USA) following the approach proposed by Zhang et al. [21]. A mixture of the lyophilized sample (1 mg) and dried potassium bromide (100 mg) was ground into a fine powder, which was then pressed into a thin slice. For each analysis, 32 scans were performed at an interval of 4 cm^−1^ between 4000 cm^−1^ and 400 cm^−1^, and the absorbance of infrared radiation was determined. Peakfit Software (version 4.12) was used for the curve fitting.

### 2.6. Free Amino Acids Determination

The concentrations of free amino acids in rice sourdough samples were measured using an automated amino acid analyzer (L-8900, Hitachi High Technologies Corp., Tokyo, Japan), following the method reported by Fu et al. [23]. Briefly, the freeze-dried sourdough powder (50 mg) was hydrolyzed with 10 mL of 6 mol/L HCl in a sealed-vacuum ampoule at 110 °C for 22 h. The reaction vial was cooled and vacuum-dried to remove the HCl. The residue was redissolved in 1 mL of 0.2 mol/L sodium citrate buffer (pH 2.2), filtered through a 0.22 μm filter, and then analyzed with the automated amino acid analyzer using manufacturer standard protocols.

### 2.7. Steamed Rice Bread Manufacture

To prepare the dough, rice sourdough (60 g) was mixed with 120 g rice flour, 60 g water, and 1.5 g yeast (Angel Yeast, Shanghai, China), kneaded for 3 min, and fermented at 30 °C for 1 h at 80% RH. The contents of the ingredients were chosen through preliminary experiments, and the formulations for steamed rice bread are shown in Table 1. The rice dough was manually shaped and proofed for 30 min at the same temperature and humidity. Finally, steaming was performed for 20 min. The same method was used for the control preparation, but the rice sourdough was replaced with 30 g rice flour and 30 g water. All SRBs were cooled for 2 h at ambient temperature before further analysis. The experiments were performed in triplicate.

### 2.8. Specific Volume and Texture Profile Analyses

The sample was weighed, and the rapeseed displacement method (AACC method 2000) was used to determine the volume. The specific volume was expressed as mL/g and was assessed by dividing the volume by the weight. The SRBs’ textural characteristics were estimated using a TA-XT texture analyzer (TMS-PRO, Food Technology Corporation, Sterling, VA, USA) in Texture Profile Analysis (TPA) mode. Each sample was uniformly sliced to a thickness of 15 mm and compressed to 40% deformation at the center using a cylindrical probe with a diameter of 75 mm. The setting parameters were: a 2 mm/s pre-test speed, 1 mm/s test speed, 5 mm/s post-test speed, 5 g trigger force, and two compression cycles at intervals of 5 s.

### 2.9. Analysis of Volatile Compounds in Steamed Rice Bread

#### 2.9.1. Gas Chromatography-Ion Mobility Spectrometry (GC-IMS) Analysis

The GC-IMS of SRBs was carried out using the GC-IMS instrument (Flavourspec^®^, G.A.S, Dortmund, Germany), as per the protocol described of Rong et al. [24] with slight modifications. Samples (3 g) were cut into pieces of approximately 2 × 2 × 2 mm and placed in 20 mL headspace vials, followed by incubation at 60 °C for 10 min. Subsequently, the headspace gas (500 μL) was automatically administered into the injection port using a syringe heated to 85 °C. Chromatographic separation was performed on a polar capillary column (WAX, 30 m × 53 mm × 1 μm; RESTEK Company, Bellefonte, PA, USA), with the column temperature maintained at 60 °C. The carrier gas was nitrogen (purity ≥ 99.999%). The programmed flow was: 2 mL/min for 2 min, 10 mL/min for 10 min, 100 mL/min for 15 min, and 150 mL/min for 20 min. The ions obtained were then driven to the migration tube, which was kept at 45 °C. The drift of the N_2_ gas flow was maintained at 150 mL/min. The retention index (RI) of the volatile compounds within the SRB samples was assessed using N-ketones, C4–C9, as external references. Volatile compounds were identified by comparing the drift time and RI with those of standard substances in both the IMS database and NIST library. The preliminary choice and analysis of the GC-IMS data were carried out using Laboratory Analytical Viewer (LAV) software (version 2.0.0).

#### 2.9.2. Gas Chromatography-Mass Spectrometry (GC-MS) Analysis

The volatile compounds in the SRB samples were also analyzed using solid-phase microextraction gas chromatography coupled with mass spectrometry, following the methods reported by Yang et al. [18]. The sample (3 g) was placed in a 20 mL headspace vial, 1 g of NaCl was added, and the vial was sealed. A 75-μm Carboxen/polydimethylsiloxane (CAR/PDMS; Supelco, Bellefonte, PA, USA) fiber was inserted into the headspace vial at 60 °C. After 30 min of extraction, the fiber was inserted into the injection port of the gas chromatograph (7890B; Agilent Technologies, Santa Clara, CA, USA), attached with a DB-WAX capillary column (30 m long, 0.25 mm internal diameter, and 0.25 μm film thickness, J&W Scientific, Folsom, CA, USA), to desorb the extracted volatiles at 250 °C for 4 min in splitless mode. Initially, the temperature of the column was maintained at 40 °C for 2 min, followed by an increase to 230 °C at a rate of 5 °C/min. The carrier gas (High-purity helium) was issued at a flow rate of 1 mL/min. MS was performed on an MS detector (5977A; Agilent Technologies) in scan mode (35–500 *m*/*z*) at 70 eV. Volatile compounds with a similarity score > 80% were identified by comparing the mass spectral data to those in the NIST 17 library.

### 2.10. Statistical Analysis

Six replicates of texture profile analysis were conducted, whereas other analyses were conducted in triplicate. One-way ANOVA analysis and Tukey’s multiple-range tests were conducted using IBM SPSS Statistics version 26. *p* < 0.05 was considered statistically significant. Principal component analysis (PCA) was applied to compare SRBs fermented with different LAB strains based on their volatile compounds using Canoco 5 (version 5.03).

## 3. Results and Discussion

### 3.1. pH and TTA Determination in Sourdough Samples

**Figure 1:**
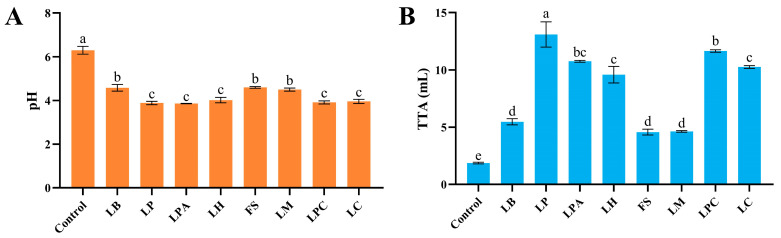
pH (**A**) and TTA (**B**) of rice sourdough fermented by different LAB strains and Control (without LAB) after 12 h. Different letters represent statistically significant differences (*p* < 0.05).

Acidification is a key feature of sourdough fermentation, and a drop in pH can stimulate proteases and amylases, which substantially affect the end products’ flavor and quality [4]. All LAB strains acidified rice dough to different degrees (Figure 1). The pH values of the different LAB fermented rice sourdoughs after 12 h were 3.86 ± 0.01 (LPA) to 4.60 ± 0.04 (FS), which were substantially lower than that of the control. The same findings were reported in a previous study in which LAB strains fermented wheat dough [25]. Simultaneously, TTA increased, corresponding to a decrease in pH. The TTA values of rice sourdoughs fermented with LABs were substantially higher than those of the control, at 4.57 ± 0.25 (FS) to 13.10 ± 1.10 (LP). During sourdough fermentation, LAB can produce a large number of organic acids, which lower the pH and increase the TTA of the rice sourdoughs [17]. The *L. plantarum*-fermented sourdough had the highest TTA value after 12 h, indicating a relatively high acidifying activity of *L. plantarum*, which is consistent with a previous study [25]. This can be attributed to the relatively large genome size of *L. plantarum*, which allows for its excellent ecological fitness and longer fermentation in the sourdough environment [26,27]. In contrast, sourdough fermented with *F. sanfranciscensis*, *L. mindensis*, and *L. brevis* had substantially lower TTA and higher pH values than those fermented with the other strains, indicating that these three LAB strains exhibited lower capacities to produce organic acids during rice sourdough fermentation.

### 3.2. SDS-PAGE Analysis

**Figure 2:**
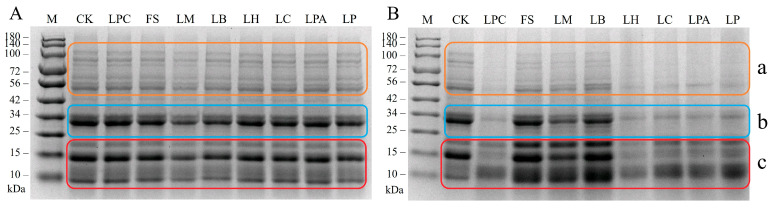
SDS-PAGE analysis of rice sourdoughs fermented with different LAB strains for 0 h (**A**) and 12 h (**B**). M, molecular weight standard proteins; CK, the control rice dough (without LAB).

To investigate the effect of LAB fermentation on the protein subunits in rice sourdough, SDS-PAGE analysis was conducted. As shown in Figure 2, all LAB strains affected protein hydrolysis to different extents. After 12 h of fermentation, obvious decreases in the intensity of protein bonds (parts a, b, and c) were observed in samples LPC, LH, LC, LPA, and LP compared to the control, indicating that more protein hydrolysis occurred in rice sourdoughs fermented with these strains. Conversely, the FS, LM, and LB samples exhibited only slight protein hydrolysis during fermentation. The intensity of large molecule protein bands (80~100 kDa) was reduced a little in these three samples after 12 h of fermentation, whereas no marked changes existed in the lower protein bands (56 kDa and part b, *Mw* ≈ 34 kDa). An acidic environment can induce protein hydrolysis by activating the cereal endogenous proteinases [4,28] and increasing the solubility and swelling of gluten proteins [29]. The lower capacities of strains FS, LM, and LB to produce organic acids may explain the slight changes in the intensity of the protein bands during sourdough fermentation (Figure 1). It is noteworthy that the intensities of the 20 kDa and 10 kDa protein bands were markedly higher in the FS, LM, and LB samples than in the control and other samples. After primary proteolysis, LAB hydrolyze peptides via various intracellular peptidases to release free amino acids [30]. The accumulation of these low molecular proteins in the FS, LM, and LB samples might indicate relatively low peptidase activity in these LAB strains during rice sourdough fermentation.

### 3.3. Fourier Infrared (FT-IR) Analysis

**Figure 3:**
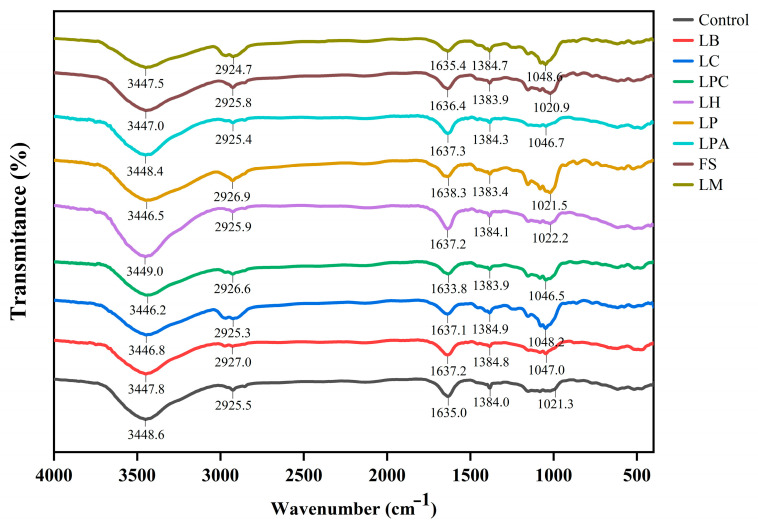
Representative FT-IR spectra of rice sourdoughs fermented by different LAB strains and Control (without LAB) after 12 h.

Through this analysis, different chemical bonds and functional groups present in the material being tested can be identified based on the relative rotation and vibration of the molecules. The FT-IR spectra of rice sourdough samples are illustrated in Figure 3. The spectra of all rice sourdough samples were similar to the control, with four main peaks in the functional group region (4000–1200 cm^−1^) and a distinct peak in the fingerprint region (1200–600 cm^−1^) [31], indicating that no new characteristic peaks appeared after LAB fermentation. Nonetheless, slight changes were observed in the amplitudes of some of the peaks. Similar findings have been reported in which LAB strains were used to ferment buckwheat or sorghum dough [21,32]. The broad absorption peak appearing within the 3200–3600 cm^−1^ region was associated with the stretching vibrations of O–H, which is ascribed to potential interactions between water and polysaccharides such as starches [33,34]. Except for LH, the stretching vibrational signals of the hydroxyl groups generated by the samples with LAB shifted slightly to the right relative to the control. This indicates that LAB fermentation could promote the strengthening of inter- and intra-molecular hydrogen bonds and increase cross-linking of rice sourdough to some extent, but there was variation among different strains. This result was in line with the findings of Zhang et al. [21]. The weak absorption peaks at approximately 2925 cm^−1^ were caused by the stretching vibrations of the C–H bonds on saturated carbon [35]. As reported by Lin et al. [34], this peak was associated with asymmetric stretching vibrations in the methylene –CH_2_ group of aliphatic chains, and the signals of this peak generated by the samples with LB, LP, and LPC were shifted from 2925.5 cm^−1^ to 2927.0 cm^−1^, 2926.9 cm^−1^, and 2926.6 cm^−1^, respectively, relative to the control. The strong absorption peak at approximately 1635 cm^−1^ corresponded to the stretching vibrations of C=O, which is the characteristic peak of proteins and belongs to the amide I band (1600–1700 cm^−1^) [36]. Amide I is an important protein region that provides information on the secondary structure of proteins [37]. The stretching vibrational signals of this peak generated by the samples with LAB were shifted compared to those of the control, indicating modifications in the protein structure of the rice sourdough during LAB fermentation. The absorption peaks at approximately 1384 cm^−1^ were attributed to the bending of O–H [21]. In the fingerprint region, the peaks at 950–1200 cm^−1^ were characteristic peaks of polysaccharides [38], which were primarily due to polysaccharides in rice flour composition. Moreover, the peak at approximately 1047 cm^−1^ became more evident in the samples with LB, LC, LPC, and LM, showing influences on polysaccharide changes in rice sourdough fermented by these LAB strains.

### 3.4. Analysis of the Free Amino Acids in Rice Sourdoughs

Free amino acids, as microbial metabolites, substantially affect the flavor, sensory quality, and nutritional value of the final product [4]. Free amino acids in different sourdough samples after 12 h of fermentation were determined using an amino acid analyzer. Table 2 lists the 17 free amino acids found in the samples, including 7 essential amino acids and 10 nonessential amino acids. Except for LB, the total free amino acid content increased in the rice sourdoughs relative to that in the control. Essential amino acids are important factors in determining food quality. As shown in Table 2, fermentation with LAB, except for LB, increased the total content of essential amino acids, but the concentration of individual essential amino acids changed depending on the LAB used. During sourdough fermentation, amino acids are accumulated by strain-specific intracellular peptidases of lactobacilli [30], which may result in variable concentrations of essential amino acids among the samples fermented by different LAB strains. These amino acids enhance the positive effects of nutrients on the human body by coordinating and promoting their absorption [39]. Moreover, it is worth noting that samples with some LABs showed various increases in the concentration of flavor-related amino acids, including Ala, Tyr, Asp, and Glu, compared to the control. The levels of these amino acids increased in samples fermented with LPA and FS. The peptide hydrolases of *F. sanfranciscensis* have been characterized and showed the highest aminopeptidase, dipeptidase, tripeptidase, and iminopeptidase activities compared with other sourdough LAB [40], which may explain the increased content of flavor-related amino acids in the FS sample. Ala and Tyr are related to sweetness, whereas Asp and Glu are associated with umami [41]. Overall, the results indicated positive effects on rice sourdough properties, including nutritional value and flavor, during LAB fermentation.

### 3.5. Specific Volume and Textural Property Analysis of Steamed Rice Bread

**Figure 4:**
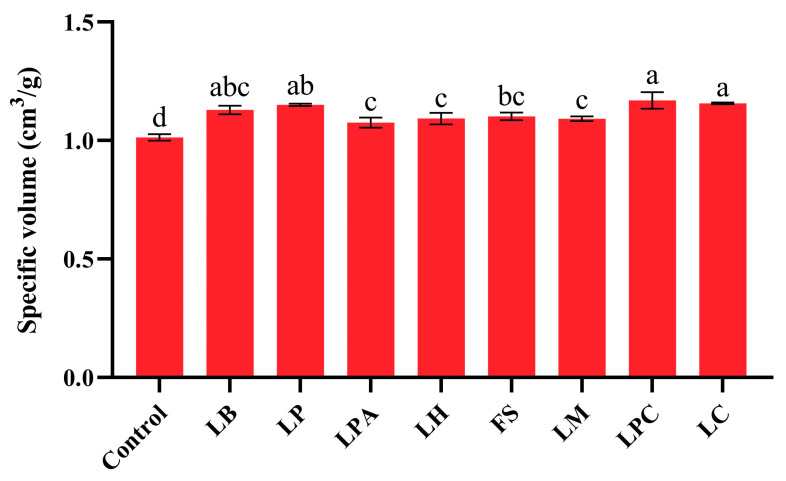
Specific volume of the SRBs based on sourdough fermentation with different LAB strains and Control (without LAB). Different letters represent statistically significant differences (*p* < 0.05).

Specific volume is a crucial characteristic index of steamed bread, and its increase benefits the quality of the final product [42,43]. The specific volumes of the samples with LABs were all significantly higher than those of the control (*p* < 0.05), indicating that LAB fermentation improved the volume of the SRBs (Figure 4). This finding is in agreement with previous studies on GF bread [44,45]. Moreover, the specific volumes varied among samples fermented with different LAB strains. As reported by Dan et al. [4], the acidification degree had different effects on the specific volume of bread. The LPC and LC groups, which had relatively low pH values in their fermented rice sourdoughs, had higher specific volumes than the other groups. However, the relatively high specific volume of the LB group did not correspond to a low pH value. In addition to acidification, EPS produced by LAB can improve the volume of steamed bread by combining with starch granules, resulting in gas retention [21,43]. Hence, the different EPS contents of rice sourdoughs may also result in different specific volumes. The specific volume of the LPC group (1.17 ± 0.04 mL/g) was highest and increased by 15.4% when compared to the control, but was still markedly lower than that of steamed wheat breads (2.55 mL/g) reported in the paper [46]. This can be attributed to the low prolamin content of rice flour, which prevents it from forming dense networks of gluten-like proteins.

The effect of LAB fermentation on the texture of the SRB was shown in Table 3. The hardness, adhesiveness, gumminess, and chewiness of the samples with LABs were lower than those of the control, whereas cohesiveness and springiness were not substantially different from those of the control. During sourdough fermentation, EPS produced by LAB can act as hydrocolloids to bind firmly to water and other dough ingredients, which retains additional moisture and results in products with low hardness [43]. Moreover, the lower hardness, adhesiveness, gumminess, and chewiness may be attributed to the enlarged specific volume of LAB-fermented SRB compared with the control [47]. The hardness value of the sample containing LC was the lowest and was reduced by 39.9% relative to the control. Hardness is the primary textural feature of steamed bread, and a lower value indicates an improvement in sensory properties [48]. The adhesiveness value is described as the negative work between two cycles in texture profile analysis [32] and was considerably lower for the LC group than for the other samples. Adhesiveness represents the stickiness of steamed bread, and a higher value implies that more food will stick to the teeth following mastication [4]. Hence, the LC group, which showed the lowest adhesiveness value, was easier for the consumers to chew and swallow. The gumminess and chewiness of the LPC group were lower than those of the other groups and were reduced by 39.1% and 37.9%, respectively, compared to the control. Gumminess and chewiness negatively affect the quality of steamed bread [47,49]. The energy required to chew food depends on chewiness, which is determined by hardness, springiness, and cohesiveness [50]. Compared to other LABs, LPC-fermented SRB had a lower chewiness value, which was compatible with its hardness values. Overall, sourdough fermentation with different LAB strains induced marked differences in the SRB’s textural properties, which is consistent with previous findings on GF sourdough bread fermented by LABs [21,32]. The SRBs fermented with LC and LPC exhibited better textural qualities, such as lower hardness, gumminess, and chewiness.

### 3.6. Volatile Compound Profiles of Steamed Rice Bread

#### 3.6.1. Volatile Compounds Analyzed by GC-IMS

**Figure 5:**
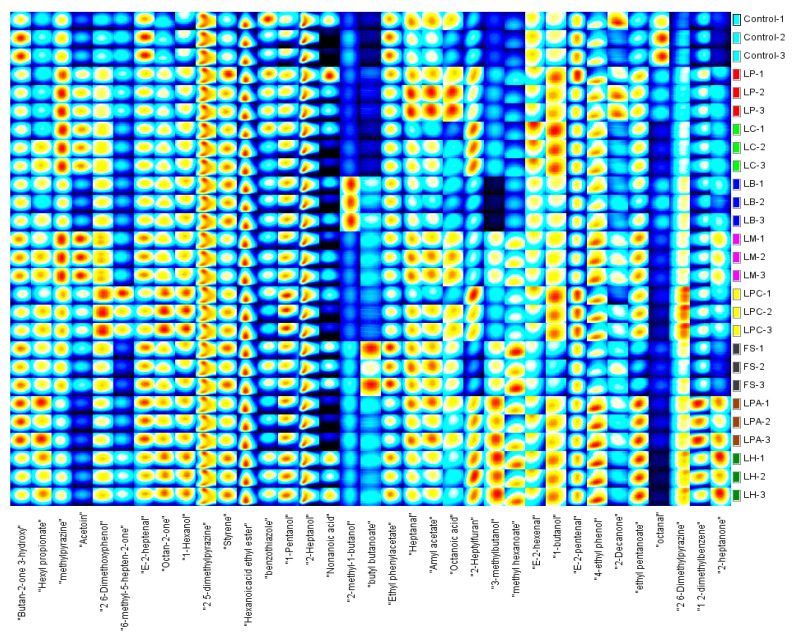
Fingerprints of volatile compounds identified in the SRBs based on sourdough fermentation with different LAB strains and Control (without LAB) by GC-IMS.

The GC-IMS technology, with its advantages of high separation capacity, extraordinary sensitivity, visualization of flavor substances, and easy operation, has been applied to detect volatile components in many types of foods [24,51,52,53]. A total of 35 volatile compounds were detected in the SRB samples using the GC-IMS NIST database, including 6 alcohols, 5 aldehydes, 6 ketones, 2 acids, 7 esters, 4 heterocyclic compounds, 3 benzene derivatives, and 2 phenols (Figure 5). Figure 5 and Table 4 describe clear variations in the peak intensities of volatile compounds among the SRB samples. The contents of 2,6-dimethoxyphenol, octan-2-one, 1,2-dimethylbenzene, and 2-heptanone were substantially higher in the samples fermented with LAB strains than in the control. Furthermore, the concentrations of volatile compounds varied in the different LAB strains fermented SRBs. The LPA and LH groups exhibited the highest peak intensities for 3-methylbutanol, hexyl propionate, ethyl pentanoate, methyl hexanoate, 1,2-dimethylbenzene, 2-heptylfuran, and 2-heptanone. The 3-methylbutanol and ethyl pentanoate are positively associated with bread aroma [54]. The contents of 2,6-dimethoxyphenol, 6-methyl-5-hepten-2-one, octan-2-one, 1-pentanol, E-2-pentenal, and 2,6-dimethylpyrazine were higher in the LPC group than in the other groups, whereas the LM group showed the highest peak intensity of acetoin, ethyl pentanoate, and 4-ethyl phenol. The esters in the FS group, including butyl butanoate, ethyl phenylacetate, and amyl acetate, had higher concentrations than other raw samples, giving the SRB fruity and floral aromas [53,54]. In contrast, the LB and LC groups had the lowest levels of volatile compounds of all the LAB strain-fermented SRBs.

#### 3.6.2. GC-MS Analysis of Volatile Compounds

**Figure 6:**
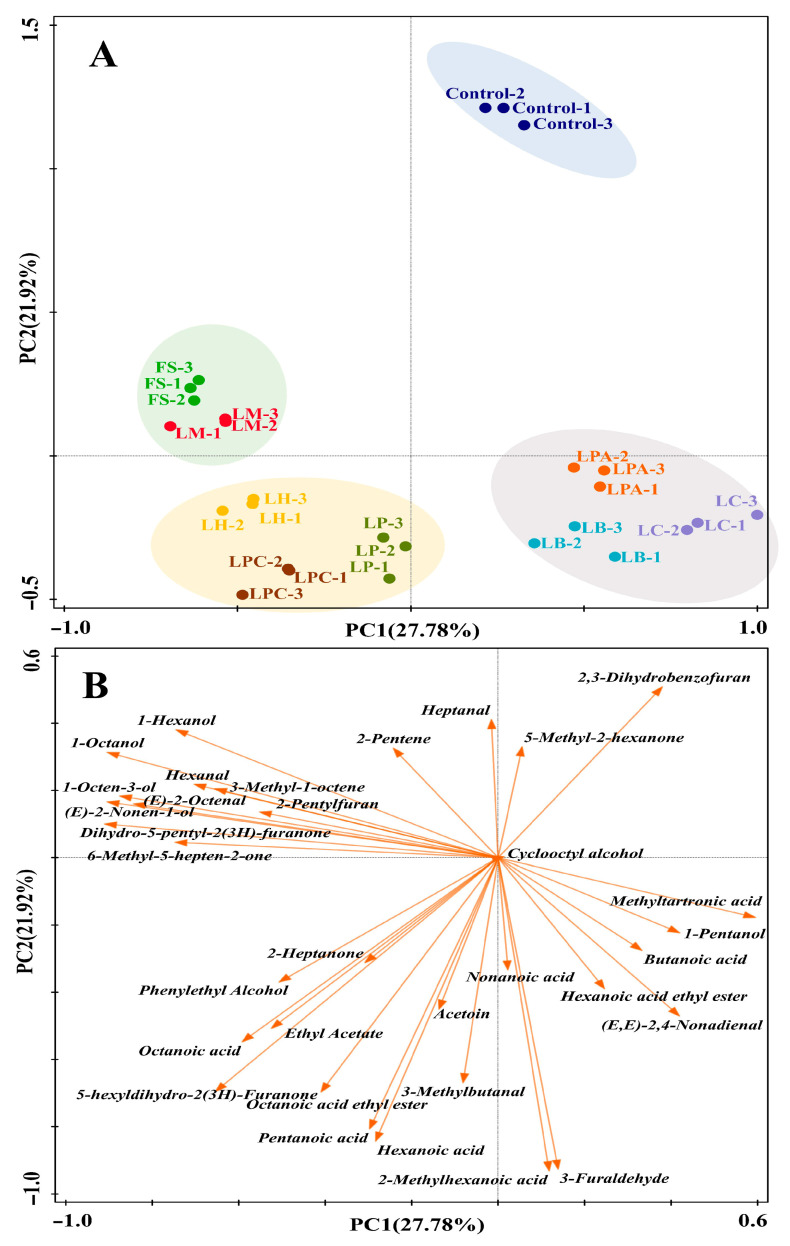
The PCA score (**A**) and loading (**B**) biplot of the SRBs fermented with different LAB strains and Control (without LAB) based on their volatile compounds identified by GC-MS.

The volatile profiles of the LAB strain-fermented SRBs and the control were also evaluated using GC-MS. A total of 33 volatile compounds were identified, including 7 alcohols, 6 aldehydes, 6 ketones, 7 acids, 3 esters, 2 heterocyclic compounds, and 2 hydrocarbons. PCA was conducted to compare the variations in the volatile profiles of the SRB samples (Figure 6). The first two principal components (PCs) explained 49.7% of the total variance. The samples were categorized into four distinct groups (Figure 6A). The loadings of the 33 volatile compounds on PC1 and PC2 were plotted in Figure 6B to reveal the characteristic volatile compounds in each group. The control, located in the first quadrant, was well separated from the samples fermented with the LAB strains in the score plot. This result demonstrated the great influence of LAB fermentation on the SRBs’ volatile profiles. The fermented samples from the FS and LM strains clustered together in the second quadrant and featured relatively high concentrations of 1-hexanol, 1-octanol, 1-octen-3-ol, hexanal, (E)-2-octenal, 2-pentylfuran, (E)-2-nonen-1-ol, dihydro-5-pentyl-2(3H)-furanone, and 6-methyl-5-hepten-2-one. All these compounds have been identified in Chinese steamed bread [25,55]. The 1-hexanol, (E)-2-octenal, 1-octen-3-ol, and hexanal are considered to be derived from lipid oxidation catalyzed by lipoxygenase [54]. Nevertheless, it is worth noting that their content can also be affected by LAB [25]. Of these compounds, 1-octen-3-ol, hexanal, and 1-hexanol are negatively associated with bread aroma [56,57]. The sample fermented with FS had both the highest concentration of hexanal and 1-hexanol, which appeared to contradict the theory that 1-hexanol in sourdough was converted from hexanal [58]. The reason for this may be that *F. sanfranciscensis* could produce 1-hexanol through other pathways or facilitate the production of hexanal from lipid oxidation in sourdough fermentation [54]. The compound 2-pentylfuran, with butter, green bean, and floral flavors, could originate from Maillard reactions or be generated by fermentation [59,60]. Moreover, sourdough fermentation also has an impact on Maillard volatile compound production, as the LAB-mediated low pH is suitable for initiating Maillard reactions [54]. Dihydro-5-pentyl-2(3H)-furanone (γ-nonalactone), which was first reported in Chinese steamed bread by Liu et al. [55], was identified in all samples of SRB in the current study. This compound, the content of which was highest in the sample fermented with LM, possesses a “coconut-like, sweet, fruity” odor [61,62]. The group located in the third quadrant consisted of the three samples fermented with LP, LPC, and LH. This group was distinguished by relatively high concentrations of ketones and acids, including 2-heptanone, 5-hexyldihydro-2(3H)-furanone, acetoin (3-hydroxy-2-butanone), pentanoic acid, hexanoic acid, and octanoic acid, as well as phenylethyl alcohol and certain esters, such as ethyl acetate and octanoic acid ethyl ester. Ketones, another group of carbonyl compounds that may stem from lipid oxidation, could also be affected by LAB in sourdough fermentation [25]. Acetoin and phenylethyl alcohol, the contents of which were highest in the LP and LPC fermented samples, respectively, have been reported to be positively correlated with bread aroma [54]. Acetoin, as a Maillard compound, can also be produced by LAB [25], which may explain its varying concentrations among the SRB samples. Ethyl acetate and octanoic acid ethyl ester are two typical esters formed by the reaction between alcohols and acetyl coenzyme A derivatives of fatty acids [62]. They are considered important flavor ingredients in steamed bread because they possess sweet, fruity, and pleasant odors [59]. The samples fermented with LPA, LB, and LC were grouped in the fourth quadrant and were characterized by relatively high contents of methyltartronic acid, 1-pentanol, butanoic acid, hexanoic acid ethyl ester, and (E, E)-2,4-nonadienal. This group exhibited fewer explicit explanatory compounds in the biplot than the groups located in the second and third quadrants, which seemed to have relatively balanced volatile profiles. The 1-pentanol and (E, E)-2,4-nonadienal were generated by lipid oxidation reactions [54,63]. Butanoic acid, with a “sweaty, rancid” odor [64], has been found to negatively influence bread aroma [65].

## 4. Conclusions

This study investigated the impact of different LAB strains isolated from Chinese traditional sourdoughs on rice sourdough properties and the quality of SRB. Among them, *L. plantarum* demonstrated the highest acidifying activity. Strains LPC, LH, LC, LPA, and LP showed considerable protein hydrolysis during rice sourdough fermentation and also remarkably increased the free amino acid contents in rice sourdoughs relative to the control. The FT-IR results indicated that LAB fermentation promoted the strengthening of inter- and intra-molecular hydrogen bonds and increased cross-linking in rice sourdough to some extent, but this effect varied among the different strains. Moreover, modifications in protein structures could also occur in rice sourdough during LAB fermentation. Fermentation treatment by LAB improved the volume and textural properties of SRBs. Notably, the SRBs fermented with LC and LPC had higher specific volumes and better textures. The flavor profiles of the SRB fermented by LAB strains were characterized using both GC-IMS and GC-MS. A total of 35 and 33 volatile compounds were detected by GC-IMS and GC-MS, respectively. PCA effectively differentiated the SRBs fermented by different LAB strains based on volatile profiles obtained from GC-MS. In conclusion, this study provides valuable contributions to the field of gluten-free manufacturing and highlights LAB fermentation as a promising, clean-label strategy for improving rice-based products. In future studies, proteomic and transcriptomic analyses should be conducted to better elucidate the role of LAB strains in rice sourdough fermentation.

## Figures and Tables

**Table 1:** Formulation for steamed rice bread.

Ingredients	SRB with LAB	Control
Sourdough (g)	60	0
Rice flour (g)	120	150
Water (g)	60	90
Yeast (g)	1.5	1.5

**Table 2:** The free amino acid content in the rice sourdoughs fermented by different LABs and Control (without LAB) after 12 h.

Amino Acid Content (mg/100 g)		Control	LB	LC	LPC	LH	LM	LP	LPA	FS
Essential amino acid	Thr	3.43 ± 0.09 ^a^	3.57 ± 0.04 ^a^	3.76 ± 0.06 ^a^	3.66 ± 0.32 ^a^	3.62 ± 0.21 ^a^	3.63 ± 0.08 ^a^	3.73 ± 0.25 ^a^	3.60 ± 0.17 ^a^	3.63 ± 0.12 ^a^
	Val	1.23 ± 0.10 ^b^	1.32 ± 0.01 ^ab^	1.37 ± 0.04 ^ab^	1.66 ± 0.22 ^a^	1.55 ± 0.22 ^ab^	1.52 ± 0.21 ^ab^	1.50 ± 0.09 ^ab^	1.65 ± 0.18 ^ab^	1.60 ± 0.05 ^ab^
	Met	1.62 ± 0.09 ^a^	1.57 ± 0.03 ^a^	1.65 ± 0.05 ^a^	1.62 ± 0.20 ^a^	1.60 ± 0.03 ^a^	1.60 ± 0.09 ^a^	1.58 ± 0.09 ^a^	1.63 ± 0.09 ^a^	1.60 ± 0.02 ^a^
	Ile	4.52 ± 0.02 ^ab^	4.33 ± 0.07 ^ab^	4.30 ± 0.16 ^ab^	4.63 ± 0.14 ^a^	4.56 ± 0.12 ^ab^	4.27 ± 0.14 ^b^	4.47 ± 0.21 ^ab^	4.33 ± 0.09 ^ab^	4.27 ± 0.07 ^b^
	Leu	8.27 ± 0.19 ^f^	8.99 ± 0.26 ^e^	10.23 ± 0.12 ^abc^	9.66 ± 0.28 ^cd^	9.46 ± 0.21 ^de^	10.00 ± 0.09 ^bcd^	10.32 ± 0.36 ^ab^	10.57 ± 0.12 ^ab^	10.84 ± 0.17 ^a^
	Phe	6.23 ± 0.11 ^c^	6.47 ± 0.19 ^bc^	6.33 ± 0.33 ^c^	6.66 ± 0.19 ^abc^	6.99 ± 0.02 ^a^	7.03 ± 0.19 ^a^	6.88 ± 0.18 ^ab^	6.92 ± 0.03 ^ab^	7.01 ± 0.19 ^a^
	Lys	2.80 ± 0.02 ^ab^	2.66 ± 0.02 ^b^	2.90 ± 0.08 ^ab^	3.03 ± 0.25 ^a^	2.96 ± 0.03 ^ab^	2.87 ± 0.08 ^ab^	2.76 ± 0.12 ^ab^	2.65 ± 0.11 ^b^	2.69 ± 0.10 ^b^
Total content		28.10 ± 0.51 ^c^	28.90 ± 0.44 ^bc^	30.53 ± 0.52 ^ab^	30.91 ± 1.56 ^ab^	30.74 ± 0.83 ^ab^	30.92 ± 0.86 ^ab^	31.23 ± 0.87 ^ab^	31.35 ± 0.77 ^a^	31.63 ± 0.34 ^a^
Nonessential amino acid	Asp	5.23 ± 0.20 ^d^	5.56 ± 0.08 ^cd^	5.43 ± 0.07 ^cd^	6.24 ± 0.21 ^a^	5.75 ± 0.11 ^bc^	5.30 ± 0.18 ^d^	6.33 ± 0.09 ^a^	6.45 ± 0.18 ^a^	6.05 ± 0.07 ^ab^
	Ser	5.46 ± 0.08 ^abc^	5.62 ± 0.09 ^abc^	5.53 ± 0.12 ^abc^	5.57 ± 0.11 ^abc^	5.33 ± 0.19 ^bc^	5.63 ± 0.11 ^abc^	5.85 ± 0.26 ^a^	5.72 ± 0.09 ^ab^	5.27 ± 0.08 ^c^
	Glu	34.23 ± 2.02 ^b^	36.65 ± 1.00 ^b^	41.33 ± 1.77 ^a^	42.03 ± 1.55 ^a^	44.66 ± 0.65 ^a^	41.66 ± 0.79 ^a^	42.58 ± 1.30 ^a^	42.62 ± 0.87 ^a^	44.07 ± 0.74 ^a^
	Gly	5.13 ± 0.10 ^cd^	4.99 ± 0.13 ^d^	5.36 ± 0.40 ^bcd^	5.70 ± 0.28 ^abc^	5.90 ± 0.04 ^ab^	5.82 ± 0.17 ^ab^	6.03 ± 0.25 ^a^	5.74 ± 0.09 ^ab^	5.67 ± 0.14 ^abc^
	Ala	4.23 ± 0.02 ^d^	4.65 ± 0.06 ^abc^	4.26 ± 0.28 ^cd^	4.52 ± 0.11 ^abcd^	4.36 ± 0.16 ^bcd^	4.66 ± 0.09 ^abc^	4.63 ± 0.22 ^abcd^	4.73 ± 0.07 ^ab^	4.85 ± 0.11 ^a^
	Cys	5.36 ± 0.08 ^bc^	5.46 ± 0.08 ^abc^	5.33 ± 0.13 ^c^	5.53 ± 0.19 ^abc^	5.66 ± 0.12 ^abc^	5.79 ± 0.20 ^a^	5.66 ± 0.16 ^abc^	5.83 ± 0.08 ^a^	5.72 ± 0.04 ^ab^
	Tyr	3.89 ± 0.02 ^c^	4.23 ± 0.08 ^abc^	3.96 ± 0.15 ^c^	4.23 ± 0.21 ^abc^	4.07 ± 0.09 ^bc^	4.11 ± 0.09 ^bc^	4.23 ± 0.21 ^abc^	4.56 ± 0.11 ^a^	4.37 ± 0.03 ^ab^
	His	3.10 ± 0.11 ^a^	2.99 ± 0.19 ^a^	2.90 ± 0.13 ^a^	2.80 ± 0.10 ^a^	2.92 ± 0.09 ^a^	2.93 ± 0.09 ^a^	2.90 ± 0.22 ^a^	2.89 ± 0.09 ^a^	2.91 ± 0.04 ^a^
	Arg	3.80 ± 0.03 ^d^	4.67 ± 0.07 ^ab^	4.26 ± 0.22 ^bc^	4.17 ± 0.13 ^cd^	4.48 ± 0.25 ^abc^	4.32 ± 0.12 ^abc^	4.43 ± 0.22 ^abc^	4.75 ± 0.10 ^a^	4.57 ± 0.08 ^abc^
	Pro	14.23 ± 1.02 ^a^	13.66 ± 2.01 ^a^	12.66 ± 0.33 ^a^	13.46 ± 0.19 ^a^	13.70 ± 0.69 ^a^	13.45 ± 0.38 ^a^	13.67 ± 0.21 ^a^	13.42 ± 0.22 ^a^	13.42 ± 0.23 ^a^
Total content		84.67 ± 2.87 ^c^	88.47 ± 2.82 ^bc^	91.00 ± 3.21 ^abc^	94.23 ± 2.32 ^ab^	96.81 ± 2.17 ^a^	93.67 ± 1.19 ^ab^	96.29 ± 2.37 ^a^	96.71 ± 1.15 ^a^	96.88 ± 1.18 ^a^
Total content (Essential and nonessential amino acid)		112.77 ± 3.34 ^c^	117.37 ± 3.21 ^bc^	121.54 ± 3.73 ^ab^	125.14 ± 3.88 ^ab^	127.56 ± 3.00 ^a^	124.58 ± 2.04 ^ab^	127.53 ± 3.22 ^a^	128.06 ± 1.92 ^a^	128.51 ± 1.50 ^a^

Values are means of triplicates ± SD. Values within the same row with different superscript letters are significantly different (*p* < 0.05).

**Table 3:** Textural characteristics of the SRB samples.

Samples	Hardness (N)	Adhesiveness (N.mm)	Cohesiveness	Springness (mm)	Gumminess (N)	Chewiness (mJ)
Control	169.51 ± 7.36 ^a^	24.32 ± 1.39 ^a^	0.63 ± 0.05 ^a^	3.08 ± 0.13 ^a^	100.36 ± 8.77 ^a^	331.27 ± 19.88 ^a^
LP	126.89 ± 8.89 ^b^	11.57 ± 1.77 ^df^	0.63 ± 0.05 ^a^	3.38 ± 0.15 ^a^	74.48 ± 3.30 ^bc^	224.73 ± 18.04 ^cd^
LC	101.89 ± 14.86 ^d^	2.89 ± 1.51 ^g^	0.62 ± 0.04 ^a^	3.40 ± 0.10 ^a^	67.16 ± 4.78 ^cd^	231.57 ± 17.56 ^cd^
LB	135.92 ± 6.87 ^b^	8.69 ± 0.40 ^f^	0.62 ± 0.04 ^a^	3.26 ± 0.17 ^a^	84.51 ± 6.42 ^b^	297.67 ± 36.53 ^b^
LM	136.49 ± 18.40 ^b^	19.19 ± 3.82 ^b^	0.63 ± 0.05 ^a^	3.28 ± 0.22 ^a^	80.34 ± 8.67 ^b^	251.93 ± 15.55 ^c^
LPC	106.84 ± 6.92 ^cd^	14.18 ± 1.82 ^cd^	0.60 ± 0.00 ^a^	3.34 ± 0.19 ^a^	61.13 ± 4.23 ^d^	205.73 ± 4.25 ^d^
FS	121.31 ± 8.56 ^bc^	15.82 ± 0.65 ^c^	0.65 ± 0.05 ^a^	3.09 ± 0.09 ^a^	79.03 ± 6.49 ^b^	243.07 ± 17.18 ^c^
LPA	133.47 ± 1.48 ^b^	10.46 ± 2.58 ^f^	0.63 ± 0.05 ^a^	3.08 ± 0.25 ^a^	83.45 ± 3.24 ^b^	243.97 ± 6.09 ^c^
LH	122.83 ± 9.08 ^bc^	13.45 ± 6.13 ^cd^	0.60 ± 0.00 ^a^	3.20 ± 0.30 ^a^	76.87 ± 11.85 ^bc^	247.40 ± 14.10 ^c^

Values are means of triplicates ± SD. Values within the same column with different superscript letters are significantly different (*p* < 0.05).

**Table 4:** Volatile compounds identified in the SRBs by GC-IMS.

No.	Compounds	Rt (Sec)	Peak Intensities								
			Control	LP	LC	LB	LM	LPC	FS	LPA	LH
	**Alcohols**										
1	1-Hexanol	664.13	102.67 ± 0.58 ^d^	103.33 ± 4.93 ^d^	107.33 ± 1.53 ^cd^	122.33 ± 3.21 ^b^	112.67 ± 3.79 ^c^	123.67 ± 5.86 ^b^	131.00 ± 4.36 ^a^	125.67 ± 0.58 ^ab^	123.00 ± 1.00 ^b^
2	1-Pentanol	458.43	269.33 ± 29.77 ^c^	272.67 ± 12.74 ^c^	314.00 ± 11.27 ^ab^	325.67 ± 8.14 ^ab^	309.33 ± 17.93 ^b^	349.00 ± 38.12 ^a^	314.00 ± 9.54 ^ab^	344.67 ± 4.16 ^ab^	319.33 ± 11.02 ^ab^
3	2-Heptanol	466.20	1299.67 ± 43.89 ^a^	1202.67 ± 56.01 ^bc^	1248.67 ± 11.02 ^ab^	1221.00 ± 28.62 ^bc^	1216.33 ± 9.29 ^bc^	1201.33 ± 47.08 ^bc^	1184.33 ± 24.85 ^c^	1250.33 ± 11.02 ^ab^	1159.67 ± 26.31 ^c^
4	2-Methyl-1-butanol	360.04	21.33 ± 2.08 ^d^	18.67 ± 3.79 ^d^	19.67 ± 1.53 ^d^	55.33 ± 0.58 ^a^	23.33 ± 1.53 ^d^	20.33 ± 2.52 ^d^	36.33 ± 5.51 ^b^	32.33 ± 2.08 ^bc^	30.33 ± 3.79 ^c^
5	3-Methylbutanol	236.25	134.67 ± 2.31 ^ef^	188.00 ± 23.43 ^e^	183.67 ± 9.29 ^e^	80.33 ± 6.66 ^f^	341.00 ± 45.04 ^c^	247.00 ± 4.36 ^d^	396.67 ± 71.60 ^bc^	421.00 ± 27.73 ^ab^	442.67 ± 28.31 ^a^
6	1-Butanol	242.66	158.67 ± 9.29 ^a^	108.00 ± 12.17 ^b^	111.00 ± 6.08 ^b^	155.33 ± 12.74 ^a^	114.33 ± 23.97 ^b^	106.67 ± 4.93 ^b^	146.67 ± 21.39 ^a^	114.33 ± 8.39 ^b^	107.67 ± 3.21 ^b^
	**Aldehydes**										
7	E-2-heptenal	723.56	276.33 ± 22.85 ^a^	226.67 ± 2.08 ^c^	212.67 ± 6.66 ^c^	226.67 ± 3.79 ^c^	251.67 ± 0.58 ^b^	214.33 ± 12.42 ^c^	270.33 ± 5.51 ^a^	265.67 ± 0.58 ^ab^	229.00 ± 1.73 ^c^
8	Heptanal	278.04	188.33 ± 10.69 ^d^	243.33 ± 2.08 ^c^	227.00 ± 29.51 ^cd^	255.00 ± 7.00 ^bc^	292.67 ± 12.42 ^ab^	302.00 ± 48.51 ^a^	310.0 0 ± 23.43 ^a^	320.67 ± 22.23 ^a^	290.00 ± 12.17 ^ab^
9	E-2-hexenal	249.06	141.00 ± 2.65 ^cd^	198.00 ± 28.62 ^b^	214.00 ± 6.08 ^ab^	135.00 ± 2.65 ^d^	163.00 ± 16.46 ^c^	213.33 ± 11.85 ^ab^	129.67 ± 17.04 ^d^	203.33 ± 4.93 ^b^	230.00 ± 4.36 ^a^
10	E-2-pentenal	184.06	1972.67 ± 79.40 ^abc^	1973.00 ± 109.12 ^abc^	1945.33 ± 10.79 ^bc^	1927.33 ± 74.20 ^bc^	1853.00 ± 71.04 ^c^	2091.00 ± 13.89 ^a^	1976.67 ± 117.50 ^abc^	2032.33 ± 3.79 ^ab^	2017.67 ± 11.85 ^ab^
11	Octanal	568.47	94.33 ± 13.32 ^a^	48.00 ± 1.00 ^b^	25.33 ± 0.58 ^cde^	50.33 ± 10.12 ^b^	28.67 ± 3.79 ^cd^	21.33 ± 1.53 ^de^	33.00 ± 2.65 ^c^	25.67 ± 2.08 ^cde^	15.00 ± 1.73 ^e^
	**Ketones**										
12	Butan-2-one-3-hydroxy	872.97	177.67 ± 19.66 ^bc^	150.67 ± 0.58 ^de^	146.00 ± 2.65 ^e^	156.33 ± 6.66 ^de^	178.67 ± 1.53 ^bc^	151.00 ± 10.44 ^de^	186.33 ± 6.43 ^ab^	197.00 ± 1.00 ^a^	165.33 ± 1.53 ^cd^
13	Acetoin	753.17	213.33 ± 31.50 ^f^	420.00 ± 25.16 ^c^	467.33 ± 7.23 ^b^	229.00 ± 6.08 ^f^	515.67 ± 8.96 ^a^	368.67 ± 3.21 ^d^	303.33 ± 35.81 ^e^	178.33 ± 6.43 ^g^	218.67 ± 1.53 ^f^
14	6-Methyl-5- hepten-2-one	717.88	124.67 ± 10.12 ^d^	141.67 ± 0.58 ^c^	105.67 ± 3.21 ^ef^	100.00 ± 2.65 ^f^	114.67 ± 2.08 ^de^	212.00 ± 19.08 ^a^	81.00 ± 3.61 ^g^	103.33 ± 2.08 ^ef^	159.00 ± 1.00 ^b^
15	Octan-2-one	682.19	49.33 ± 8.96 ^e^	73.33 ± 4.16 ^d^	78.00 ± 0.00 ^d^	88.33 ± 0.58 ^c^	88.00 ± 2.65 ^c^	119.67 ± 1.53 ^a^	93.00 ± 7.00 ^c^	107.67 ± 1.53 ^b^	118.67 ± 3.21 ^a^
16	2-Decanone	192.57	41.00 ± 19.08 ^bc^	56.67 ± 2.08 ^a^	27.00 ± 0.00 ^c^	26.33 ± 3.21 ^c^	38.67 ± 1.53 ^bc^	43.33 ± 12.42 ^ab^	39.00 ± 0.00 ^bc^	47.33 ± 5.51 ^ab^	37.00 ± 1.00 ^bc^
17	2-Heptanone	190.78	19.00 ± 4.36 ^e^	40.67 ± 10.69 ^c^	30.33 ± 2.08 ^cd^	25.67 ± 0.58 ^de^	38.67 ± 5.51 ^c^	51.67 ± 3.21 ^b^	26.67 ± 6.66 ^de^	60.00 ± 7.81 ^ab^	64.00 ± 5.20 ^a^
	**Esters**										
18	Hexyl propionate	841.79	109.00 ± 7.81 ^e^	113.67 ± 4.93 ^de^	136.67 ± 23.12 ^cd^	132.00 ± 15.62 ^cde^	149.67 ± 10.12 ^bc^	149.33 ± 16.77 ^bc^	152.00 ± 0.00 ^bc^	178.67 ± 11.85 ^a^	172.33 ± 12.42 ^ab^
19	Hexanoicacid ethyl ester	506.31	1319.33 ± 131.36 ^abc^	1382.67 ± 85.17 ^abc^	1226.33 ± 187.65 ^c^	1314.33 ± 3.79 ^abc^	1281 ± 22.54 ^bc^	1271.33 ± 146.07 ^bc^	1430.33 ± 9.87 ^ab^	1260.67 ± 25.42 ^bc^	1465.00 ± 12.17 ^a^
20	Butyl butanoate	343.98	12.00 ± 1.00 ^b^	13.67 ± 0.58 ^b^	12.00 ± 1.00 ^b^	25.67 ± 1.53 ^a^	13.33 ± 0.58 ^b^	10.00 ± 1.00 ^b^	23.67 ± 6.66 ^a^	13.33 ± 2.08 ^b^	14.00 ± 2.65 ^b^
21	Ethyl phenylacetate	306.18	244.00 ± 7.00 ^b^	216.00 ± 13.89 ^c^	209.00 ± 5.29 ^c^	238.00 ± 7.00 ^b^	213.67 ± 15.04 ^c^	195.00 ± 10.44 ^c^	277.67 ± 21.39 ^a^	205.00 ± 9.54 ^c^	238.00 ± 9.54 ^b^
22	Amyl acetate	276.99	97.67 ± 1.53 ^d^	139.33 ± 3.79 ^bc^	120.33 ± 18.50 ^cd^	132.33 ± 3.79 ^c^	160.00 ± 4.36 ^ab^	163.67 ± 29.16 ^ab^	167.67 ± 10.12 ^a^	178.33 ± 15.89 ^a^	158.67 ± 9.29 ^ab^
23	Methyl hexanoate	235.09	243.33 ± 1.53 ^d^	286.33 ± 9.45 ^c^	298.00 ± 23.43 ^c^	243.00 ± 15.62 ^d^	420.67 ± 14.47 ^b^	321.00 ± 8.72 ^c^	448.67 ± 43.89 ^b^	490.67 ± 26.58 ^a^	505.67 ± 6.66 ^a^
24	Ethyl pentanoate	176.50	574.67 ± 19.66 ^c^	749.33 ± 10.79 ^b^	766.33 ± 7.57 ^b^	756.33 ± 42.74 ^b^	870.33 ± 88.92 ^a^	731.00 ± 31.19 ^b^	597.67 ± 82.85 ^c^	942.33 ± 15.31 ^a^	872.33 ± 6.43 ^a^
	**Acids**										
25	Nonanoic acid	441.42	227.67 ± 264.43 ^c^	505.67 ± 118.94 ^ab^	254.33 ± 163.40 ^bc^	299.33 ± 118.08 ^bc^	341.33 ± 96.42 ^abc^	255.33 ± 133.95 ^bc^	504.67 ± 28.31 ^ab^	157.33 ± 15.89 ^c^	569.00 ± 80.55 ^a^
26	Octanoic acid	247.80	77.00 ± 22.54 ^de^	101.00 ± 2.65 ^bc^	66.00 ± 3.61 ^e^	80.67 ± 4.93 ^cde^	108.33 ± 7.57 ^ab^	91.33 ± 21.08 ^bcd^	127.00 ± 7.81 ^a^	104.67 ± 6.66 ^b^	89.67 ± 6.66 ^bcd^
	**Heterocyclic compounds**										
27	Methylpyrazine	753.58	512.00 ± 15.62 ^b^	514.00 ± 7.81 ^ab^	493.67 ± 6.66 ^cd^	486.67 ± 9.87 ^d^	486.00 ± 8.72 ^d^	505.33 ± 7.23 ^bc^	528.67 ± 9.29 ^a^	462.67 ± 0.58 ^e^	479.33 ± 4.16 ^d^
28	2,5-Dimethylpyrazine	672.95	584.00 ± 1.00 ^a^	509.00 ± 1.00 ^ab^	376.00 ± 233.86 ^b^	554.33 ± 25.70 ^a^	490.33 ± 16.20 ^ab^	467.00 ± 28.62 ^ab^	615.67 ± 3.21 ^a^	547.33 ± 15.31 ^a^	491.00 ± 10.44 ^ab^
29	2-Heptylfuran	246.33	373.33 ± 70.47 ^b^	342.33 ± 28.31 ^b^	552.67 ± 32.35 ^a^	425.33 ± 0.58 ^b^	390.67 ± 51.39 ^b^	413.33 ± 81.13 ^b^	367.67 ± 54.28 ^b^	573.33 ± 56.89 ^a^	586.33 ± 49.08 ^a^
30	2,6-Dimethylpyrazine	597.87	48.00 ± 11.27 ^c^	53.33 ± 3.21 ^c^	48.33 ± 3.79 ^c^	71.00 ± 1.00 ^ab^	65.00 ± 0.00 ^b^	78.33 ± 3.79 ^a^	72.00 ± 7.81 ^ab^	70.67 ± 5.51 ^ab^	68.33 ± 4.62 ^ab^
	**Benzene derivatives**										
31	2,6-Dimethoxyphenol	752.85	103.33 ± 10.69 ^d^	115.33 ± 4.16 ^c^	116.00 ± 4.36 ^c^	144.00 ± 0.00 ^b^	121.67 ± 3.79 ^c^	161.33 ± 5.51 ^a^	143.33 ± 7.23 ^b^	140.67 ± 4.93 ^b^	150.67 ± 3.21 ^b^
32	Styrene	635.88	49.67 ± 0.58 ^d^	66.67 ± 6.43 ^bc^	62.00 ± 1.73 ^c^	69.00 ± 2.65 ^ab^	53.33 ± 4.93 ^d^	64.00 ± 1.00 ^bc^	73.33 ± 4.73 ^a^	64.33 ± 0.58 ^bc^	74.00 ± 4.36 ^a^
33	Benzothiazole	474.18	281.67 ± 50.52 ^a^	295.33 ± 27.43 ^a^	240.67 ± 66.98 ^a^	262.33 ± 4.93 ^a^	252.33 ± 14.15 ^a^	239.33 ± 52.55 ^a^	288.33 ± 3.21 ^a^	231.00 ± 8.72 ^a^	298.33 ± 1.53 ^a^
	**Phenols**										
34	4-Ethyl phenol	180.18	329.33 ± 17.62 ^d^	421.67 ± 93.85 ^abc^	375.00 ± 23.39 ^bcd^	422.67 ± 1.53 ^abc^	457.00 ± 45.92 ^a^	453.00 ± 8.72 ^a^	368.00 ± 38.97 ^cd^	448.00 ± 17.35 ^ab^	405.33 ± 7.23 ^abc^
35	1,2-Dimethylbenzene	169.89	305.00 ± 0.00 ^f^	375.00 ± 19.92 ^c^	340.67 ± 10.21 ^de^	315.00 ± 19.08 ^ef^	357.00 ± 4.36 ^cd^	360.00 ± 1.00 ^cd^	338.00 ± 4.36 ^de^	501.33 ± 16.77 ^a^	429.00 ± 27.71 ^b^

Rt: retention time. Values are means of triplicates ± SD. Values within the same row with different superscript letters are significantly different (*p* < 0.05).

## Data Availability

The data that support the findings of this study are available from the corresponding author upon reasonable request.

## References

[1] De Angelis M., Cassone A., Rizzello C.G., Gagliardi F., Minervini F., Calasso M., Di Cagno R., Francavilla R., Gobbetti M. (2010). Mechanism of degradation of immunogenic gluten epitopes from *Triticum turgidum* L. var. durum by sourdough lactobacilli and fungal proteases. Appl. Environ. Microbiol..

[2] Green P.H., Lebwohl B., Greywoode R. (2015). Celiac disease. J. Allergy Clin. Immunol..

[3] Turabi E., Sumnu G., Sahin S. (2008). Rheological properties and quality of rice cakes formulated with different gums and an emulsifier blend. Food Hydrocoll..

[4] Dan H., Gu Z., Li C., Fang Z., Hu B., Wang C., Chen S., Tang X., Ren Y., Wu W. (2022). Effect of fermentation time and addition amount of rice sourdoughs with different microbial compositions on the physicochemical properties of three gluten-free rice breads. Food Res. Int..

[5] Masure H.G., Wouters A.G., Fierens E., Delcour J.A. (2019). Impact of egg white and soy proteins on structure formation and crumb firming in gluten-free breads. Food Hydrocoll..

[6] Nicolae A., Radu G.-L., Belc N. (2016). Effect of sodium carboxymethyl cellulose on gluten-free dough rheology. J. Food Eng..

[7] Hager A.-S., Arendt E.K. (2013). Influence of hydroxypropylmethylcellulose (HPMC), xanthan gum and their combination on loaf specific volume, crumb hardness and crumb grain characteristics of gluten-free breads based on rice, maize, teff and buckwheat. Food Hydrocoll..

[8] Shin M., Gang D.-O., Song J.-Y. (2010). Effects of protein and transglutaminase on the preparation of gluten-free rice bread. Food Sci. Biotechnol..

[9] Bender D., Schönlechner R. (2020). Innovative approaches towards improved gluten-free bread properties. J. Cereal Sci..

[10] De Vuyst L., Neysens P. (2005). The sourdough microflora: Biodiversity and metabolic interactions. Trends Food Sci. Technol..

[11] Gobbetti M., De Angelis M., Di Cagno R., Rizzello C.G. (2008). Sourdough/lactic acid bacteria. Gluten-Free Cereal Products and Beverages.

[12] Moroni A.V., Dal Bello F., Arendt E.K. (2009). Sourdough in gluten-free bread-making: An ancient technology to solve a novel issue?. Food Microbiol..

[13] Siepmann F.B., Ripari V., Waszczynskyj N., Spier M.R. (2018). Overview of Sourdough Technology: From Production to Marketing. Food Bioprocess Technol..

[14] Galle S. (2012). Sourdough: A tool to improve bread structure. Handbook on Sourdough Biotechnology.

[15] Arendt E.K., Ryan L.A., Dal Bello F. (2007). Impact of sourdough on the texture of bread. Food Microbiol..

[16] Fan H., Zheng X., Ai Z., Liu C., Li R., Bian K. (2018). Analysis of volatile aroma components from Mantou fermented by different starters. J. Food Process. Preserv..

[17] Wang Y., Yang Y., Li H., Zhang Q., Xu F., Li Z. (2021). Characterization of aroma-active compounds in steamed breads fermented with Chinese traditional sourdough. LWT—Food Sci. Technol..

[18] Yang H., Liu T., Zhang G., He G. (2020). Intraspecific diversity and fermentative properties of *Saccharomyces cerevisiae* from Chinese traditional sourdough. LWT—Food Sci. Technol..

[19] Liu T., Li Y., Chen J., Sadiq F.A., Zhang G., Li Y., He G. (2016). Prevalence and diversity of lactic acid bacteria in Chinese traditional sourdough revealed by culture dependent and pyrosequencing approaches. LWT—Food Sci. Technol..

[20] Kline L., Sugihara T.F. (1971). Microorganisms of the San Francisco Sour Dough Bread Process: II. Isolation and Characterization of Undescribed Bacterial Species Responsible for the Souring Activity. Appl. Microbiol..

[21] Zhang J., Yao Y., Li J., Ju X., Wang L. (2023). Impact of exopolysaccharides-producing lactic acid bacteria on the chemical, rheological properties of buckwheat sourdough and the quality of buckwheat bread. Food Chem..

[22] Yang H., Sadiq F.A., Liu T., Zhang G., He G. (2020). Use of physiological and transcriptome analysis to infer the interactions between *Saccharomyces cerevisiae* and *Lactobacillus sanfranciscensis* isolated from Chinese traditional sourdoughs. LWT—Food Sci. Technol..

[23] Fu W., Wang S., Xue W. (2024). Mechanism of carbohydrate and protein conversion during sourdough fermentation: An analysis based on representative Chinese sourdough microbiota. Int. J. Food Microbiol..

[24] Rong Y., Xie J., Yuan H., Wang L., Liu F., Deng Y., Jiang Y., Yang Y. (2023). Characterization of volatile metabolites in Pu-erh teas with different storage years by combining GC-E-Nose, GC–MS, and GC-IMS. Food Chem. X.

[25] Liu T., Li Y., Yang Y., Yi H., Zhang L., He G. (2020). The influence of different lactic acid bacteria on sourdough flavor and a deep insight into sourdough fermentation through RNA sequencing. Food Chem..

[26] Molenaar D., Bringel F., Schuren F.H., de Vos W.M., Siezen R.J., Kleerebezem M. (2005). Exploring *Lactobacillus plantarum* genome diversity by using microarrays. J. Bacteriol..

[27] De Vuyst L., Van Kerrebroeck S., Harth H., Huys G., Daniel H.-M., Weckx S. (2014). Microbial ecology of sourdough fermentations: Diverse or uniform?. Food Microbiol..

[28] Yang H., Lin J., Han X., Bi J., Dong L., Sun J., Shen C., Xu Y. (2024). Functional Characterization of Different *Fructilactobacillus sanfranciscensis* Strains Isolated from Chinese Traditional Sourdoughs. Foods.

[29] Zhang Y., Hong T., Yu W., Yang N., Jin Z., Xu X. (2020). Structural, thermal and rheological properties of gluten dough: Comparative changes by dextran, weak acidification and their combination. Food Chem..

[30] Gänzle M.G., Loponen J., Gobbetti M. (2008). Proteolysis in sourdough fermentations: Mechanisms and potential for improved bread quality. Trends Food Sci. Technol..

[31] Su W.H., Sun D.W. (2018). Fourier transform infrared and Raman and hyperspectral imaging techniques for quality determinations of powdery foods: A review. Compr. Rev. Food Sci. Food Saf..

[32] Turksoy S., Guzel M., Guzel N. (2024). Effect of sourdough addition on gluten-free sorghum bread fortified with plant-based protein and dietary fiber: Functional, textural, and structural properties. Cereal Chem..

[33] Silambarasan S., Logeswari P., Cornejo P., Kannan V.R. (2019). Evaluation of the production of exopolysaccharide by plant growth promoting yeast *Rhodotorula sp.* strain CAH2 under abiotic stress conditions. Int. J. Biol. Macromol..

[34] Lin H., Bean S., Tilley M., Peiris K., Brabec D. (2021). Qualitative and quantitative analysis of sorghum grain composition including protein and tannins using ATR-FTIR spectroscopy. Food Anal. Methods.

[35] Xu X., Gao C., Xu J., Meng L., Wang Z., Yang Y., Shen X., Tang X. (2022). Hydration and plasticization effects of maltodextrin on the structure and cooking quality of extruded whole buckwheat noodles. Food Chem..

[36] Linlaud N., Ferrer E., Puppo M.C., Ferrero C. (2011). Hydrocolloid interaction with water, protein, and starch in wheat dough. J. Agric. Food Chem..

[37] Li S., Liu S., Wu H., Zhao W., Zhang A., Li P., Liu J., Yi H. (2024). Insights into the starch and proteins molecular structure changes of foxtail millet sourdough: Effect of fermentation from grains of cereal to pre-meal. Int. J. Biol. Macromol..

[38] Li J., Ai L., Xu F., Hu X., Yao Y., Wang L. (2022). Structural characterization of exopolysaccharides from Weissella cibaria NC516. 11 in distiller grains and its improvement in gluten-free dough. Int. J. Biol. Macromol..

[39] Rios M.B., Iriondo-DeHond A., Iriondo-DeHond M., Herrera T., Velasco D., Gómez-Alonso S., Callejo M.J., Del Castillo M.D. (2020). Effect of coffee cascara dietary fiber on the physicochemical, nutritional and sensory properties of a gluten-free bread formulation. Molecules.

[40] Yazar G., Tavman Ş. (2012). Functional and Technological Aspects of Sourdough Fermentation with *Lactobacillus sanfranciscensis*. Food Eng. Rev..

[41] Yang Y., Xia Y., Wang G., Tao L., Yu J., Ai L. (2019). Effects of boiling, ultra-high temperature and high hydrostatic pressure on free amino acids, flavor characteristics and sensory profiles in Chinese rice wine. Food Chem..

[42] Kim E., Lee S., Lee S. (2016). Quality characteristics of steamed rice bread prepared with different contents of proteolytic enzyme. Appl. Biol. Chem..

[43] Xu D., Hu Y., Wu F., Jin Y., Xu X., Gänzle M.G. (2020). Comparison of the functionality of exopolysaccharides produced by sourdough lactic acid bacteria in bread and steamed bread. J. Agric. Food Chem..

[44] López M.S., Susana S.L., Pérez G.T., Salvucci E.J. (2025). Enhancing the technological quality of breads with gluten-free sourdough: Application of lactic acid bacteria and yeast as lyophilized starters. Food Biosci..

[45] Chochkov R., Angelov A., Krasteva A., Rangelov I. (2025). Sorghum flour in breadmaking: Opportunities for sorghum sourdough, gluten-free bread quality and shelf life. Food Sci. Appl. Biotechnol..

[46] Xi J., Xu D., Wu F., Jin Z., Xu X. (2020). Effect of Na_2_CO_3_ on quality and volatile compounds of steamed bread fermented with yeast or sourdough. Food Chem..

[47] Wang X., Huangfu X., Zhao M., Zhao R. (2023). Chinese traditional sourdough steamed bread made by retarded sponge-dough method: Microbial dynamics, metabolites changes and bread quality during continuous propagation. Food Res. Int..

[48] Feng W., Zhang H., Wang R., Zhou X., Wang T. (2021). Modifying the internal structures of steamed rice cakes by emulsifiers for promoted textural and sensory properties. Food Chem..

[49] Tang Z., Fan J., Zhang Z., Zhang W., Yang J., Liu L., Yang Z., Zeng X. (2021). Insights into the structural characteristics and in vitro starch digestibility on steamed rice bread as affected by the addition of okara. Food Hydrocoll..

[50] Carocho M., Morales P., Ciudad-Mulero M., Fernandez-Ruiz V., Ferreira E., Heleno S., Rodrigues P., Barros L., Ferreira I.C. (2020). Comparison of different bread types: Chemical and physical parameters. Food Chem..

[51] Yang Y., Zhu H., Chen J., Xie J., Shen S., Deng Y., Zhu J., Yuan H., Jiang Y. (2022). Characterization of the key aroma compounds in black teas with different aroma types by using gas chromatography electronic nose, gas chromatography-ion mobility spectrometry, and odor activity value analysis. LWT—Food Sci. Technol..

[52] Chang X., Huang X., Tian X., Wang C., Aheto J.H., Ernest B., Yi R. (2020). Dynamic characteristics of dough during the fermentation process of Chinese steamed bread. Food Chem..

[53] Fan X., Jiao X., Liu J., Jia M., Blanchard C., Zhou Z. (2021). Characterizing the volatile compounds of different sorghum cultivars by both GC-MS and HS-GC-IMS. Food Res. Int..

[54] Pico J., Bernal J., Gomez M. (2015). Wheat bread aroma compounds in crumb and crust: A review. Food Res. Int..

[55] Liu T.J., Li Y., Sadiq F.A., Yang H.Y., Gu J.S., Yuan L., Lee Y.K., He G.Q. (2018). Predominant yeasts in Chinese traditional sourdough and their influence on aroma formation in Chinese steamed bread. Food Chem..

[56] Martínez-Anaya M.A. (1996). Enzymes and bread flavor. J. Agric. Food Chem..

[57] Paraskevopoulou A., Chrysanthou A., Koutidou M. (2012). Characterisation of volatile compounds of lupin protein isolate-enriched wheat flour bread. Food Res. Int..

[58] Kaseleht K., Paalme T., Mihhalevski A., Sarand I. (2011). Analysis of volatile compounds produced by different species of lactobacilli in rye sourdough using multiple headspace extraction. Int. J. Food Sci. Technol..

[59] Birch A.N., Petersen M.A., Hansen Å.S. (2014). Aroma of wheat bread crumb. Cereal Chem..

[60] Hansen A., Schieberle P. (2005). Generation of aroma compounds during sourdough fermentation: Applied and fundamental aspects. Trends Food Sci. Technol..

[61] Cho I.H., Peterson D.G. (2010). Chemistry of bread aroma: A review. Food Sci. Biotechnol..

[62] Birch A.N., Petersen M.A., Hansen Å.S. (2013). The aroma profile of wheat bread crumb influenced by yeast concentration and fermentation temperature. LWT-Food Sci. Technol..

[63] Paterson A., Piggott J.R. (2006). Flavour in sourdough breads: A review. Trends Food Sci. Technol..

[64] Czerny M., Schieberle P. (2002). Important aroma compounds in freshly ground wholemeal and white wheat flour identification and quantitative changes during sourdough fermentation. J. Agric. Food Chem..

[65] Quílez J., Ruiz J., Romero M. (2006). Relationships between sensory flavor evaluation and volatile and nonvolatile compounds in commercial wheat bread type baguette. J. Food Sci..

